# Selections and Abstracts

**Published:** 1916-12

**Authors:** 


### SELECTIONS AND ABSTRACTS

### SAFEGUARDING YOUR FOODS AND DRUGS

**U. S. Dept. of Agriculture.**

In the enforcement of the Food and Drugs Act during the last year, U. S. Department of Agriculture officials analyzed 29, 833 samples of foods and drugs offered for interstate shipment and for import. A physical examination was made of samples from 76,468 shipments offered for import. Of these foreign shipments, 6,353 were found to violate the law in some respects and were either excluded from the country or admitted only after the importers had relabeled them to comply with the law. Of the samples of domestic products analyzed 3,535 either because of the nature of the product or because the label on it did not tell the truth, were found to be in violation of the Federal Law. In 1,364 cases the Department recommended to the Department of Justice that criminal prosecution be instituted against the manufacturers or that the goods be seized. In many cases where there was no evidence of intention to defraud, and where there was merely some easily remedied flaw in the wording of a label, the shippers, after being warned in hearings, voluntarily took steps which made their products fully comply with the requirements. In all, there was held 8,715 such hearings, many of which resulted in the prosecutions indicated and the gathering of evidence for a large number of additional cases, which will be forwarded to the Department of Justice.

The Bureau of Chemistry, in its annual report, also calls attention to the fact that through the system of Service and Regulatory Announcements now in use, manufacturers are given due notice of the requirements and thus are enabled voluntarily to make their products conform to the law. In this way the government achieve its purposes, frequently without entering into needless and very expensive litigation.

In the regulatory work, special emphasis has been given to the control of drug products and foods liable to spoilage and pollution. These frequently constitute a serious menace to health. The food inspectors have been instructed to be particularly watchful for interstate shipments of bad eggs, milk, oysters, and spoiled canned goods, and false and fraudulently labeled medicines and spurious, synthetic drugs.

### CURBING FRAUDULENT MEDICINES

Attempts to counterfeit or adulterate imported drugs have been more common since the recent high price and scarcity of many of these products encouraged their imitation· It is interesting to note that of the 1,036 cases terminated in the courts during the year, 198 were brought on account of the false and fraudulent labeling of medicines. In all of these medical cases, save five, the courts found for the government and this ;it is believed, has exercised an important deterrent effect on the vendors of nostrums shipped from one state to another.

The work of controlling the fraudulent labels of medicines and mineral waters has been greatly strengthened by the establishment of a separate office to deal with these matters. At the request of the Secretary of Agriculture an officer of the U. S Public Health Service has been detailed to take charge of this work. Moreover, through the close cooperation established with the foods and drugs officials of many of the States, the Department was able to direct the attention of the local authorities to the presence of spurious drugs in their States and, as a result, much of these fraudulent goods in the hands of local dealers and beyond the reach of Federal authorities were destroyed by State and municipal officers who, in many cases, prosecuted those responsible for the local traffic.

### MILK, EGGS AND OYSTERS

The cooperation in the sanitary control of the milk supply of small cities described in the report for last year has been extended in Illinois, Iowa, Missouri, Kansas, Nebraska and in New England. It is proposed to repeat this work year after year, extending it each year to new territory. In some localities bad conditions were found, due in the main to insufficient cooling and careless handling. Perhaps the best results of this work has been that it stimulated some of the local authorities to take up similar work independently so that definite permanent improvement of the milk supply of a number of cities has resulted. The cooperative work on the control of the shipment of decomposed eggs described in the report of last year has been extended to cover much of the territory in which shipments originate so that eggs are now candled before shipment far more than formerly and the spoiled eggs destroyed or fed to poultry and stock. At the same time information given to local officials has helped them to curb local traffic in eggs rejected in candling.

The Bureau of Chemistry, after making cooperative sanitary surveys of oyster beds, issued warnings against the interstate shipment of oyters from polluted and doubtful beds and, where these warnings were not regarded, undertook prosecutions. As a result, interstate shipment from such territory was stopped.

**Other Adulterations.**

The campaign against the sweating of immature oranges and immature grapefruit so as to give the immature fruit the color of ripe fruit has been successful, largely because of the active help of the greater part of the citrus-fruit producers. Comparatively few sweated, immature oranges were offered during the last year, and it is believd the better quality of fruit resulted in a steadier market so that the producer as well as the consumer benefited.

Other forms of adulteation not alreody mentioned that received especial attention are the adulteration of scallops and canned tomatoes with water, the substitution of colored starch paste for tomato sauce, the reprocessing of spoiled canned goods, the traffic in cull beans, in decomposed tomato products, in rancid olive oil, in wormy horse beans, the substitution of foreign fat for cacao butter in and the addition of cacao shells to, cacao products, the adulteration of rice bran with rice hulls, the coloring of inferior macaroni and of plain noodles, the misbranding of domestic macaroni in simulation of imported goods, and the adulteration of oats with water or weed seeds.

---

### THE FAUCIAL TONSIL IN ITS MODERN ASPECT.

By JOHN J. KYLE, M.D.,
University of Southern California.
Professor of Otology and Larynology, Medical Department,

There is probably no subject possessing greater interest than the medical and surgical treatment of the faucial tonsils. There is no operation about the upper air passages that is more often performed and none requiring greater skill and aptitude on the part of the surgeon than tonsillectomy. After many years of observation I have about come to the conclusion that there is no one operation about the head that is so often poorly executed.

The development of the tonsils begins in early fetal life, about the third month, and at birth most children possess tonsils and adenoids, which are perceptible to the naked eye Not infrequently at birth children have large tonsils and adenoids, sufficient to interfere with feeding. Infants will with difficulty take the breast or the bottle, and for only a short time, crying and fretting on account of inability to breathe and take nourishment at the same time. These children are poorly nourished and do not develop naturally. Sometimes they are treated empirically for stomach or intestinal disorders, and not infrequently the nurse is requested to wash out the stomach time after time. A little careful examination will frequently disclose the source of irritation.

### Mouth Breathing.

Mouth breathing in infancy, from tonsil and adenoid tissue, predisposes to deformities of the mouth, middle-ear disease, disease of the teeth, lowering of the tissue resistance, and a variety of focal infections.

There is no age at which the tonsils and adenoids cannot be successfully removed. In very young infants, the adenoid structures are so soft that there is no particular pain connected with the removal. In young children the tonsils should be removed always under ether anesthesia.

### Function of the Tonsils.

There is nothing definite known in regard to the function of the tonsils. Many hypotheses have been advanced but none of them seem to hold water. It is argued by Briege and others that the tonsils protect the body against pathogenic infection, by passage of lymph through the tonsils to the surface. There are many reasons to think that the reverse theory is more consistent with our investigations, and that is that the tonsils are an open entrance of infection, and no tonsil that can be detected easily in a normal tonsil. Unfortunately, one can never tell, in all cases, whether a tonsil is the harborer of microorganisms or the source of systemic infection, unless they are removed and the condition of the child or adult compared some time afterward with the condition before operation.

### Anatomy.

Most medical men are more or less familiar with the anatomy of the tonsils. The position of the tonsils in their fossae surrounded by the pillars predisposes to retention of food particles, debris, and the natural secretions from the mucous membrane lining the crypts. This cannot be demonstrated by merely looking into the throat.

**Demonstrating Infections.**

If one cares to demonstrate whether or not the tonsils contain pus, cheesy deposits, or infective material, it will be necessary to cocainize and with a forcep evulse the tonsil and by making traction the tonsil may be so compressed that its contents will be squeezed out. This may be examined and the character of the infection or debris determined. Sometimes the organisms present may be the Streptococcus viridens, staphylococcus in its different varieties, pneumococcus, tubercle bacilli, diptheroid bacillus, and practically all the varieties of mouth organisms. The organisms may be confined to one or two varieties or to a very great many.

**Focal Infection.**

Pain and inflammation in the tonsils are not necessary for the establishment of focal infections. Many times the sequel of absorption from the tonsils is fairly pronounced, and the tonsils are apparently so small as to excite no attention whatever. The late Dr· Pinchon was one of the first in this country to call attention to the hyperemia or redness of the anterior pillar covering the tonsil as a reliable sign of a chronic diseased tonsil, and his observations hold good today.

The tonsils empty into the deep cervical lymphatics beneath the sterno mastoid muscle, and enlargement of the deep cervical glands of the neck is suggestive of absorption from the tonsils. There may be a simple swelling of the tonsillar lymph node, a fibrous caseous degeneration or suppuration of the gland, and which is tubercular in many cases. Sometimes in acute and chronic inflammation, the glandular enlargements in the neck disappear with local treatment of the tonsils. The tendency is for a low form of inflammation to continue for a long time, and it is during this period and before caseous degeneration or suppuration that the greatest danger to systemic infection exists. A blocking of the lymph channels by swelling of the lymph node is not sufficient to prevent the tubercle bacilli, for instance, in reaching the lung. We think it advisable, in the removal of enlarged glands of the neck, first to ascertain the possibility of tonsillar infection and remove them at the same time, thus preventing a second operation on the neck.

**The Tonsils and Tuberculosis.**

The tonsils are the most vunerable spot for the entrance of the tubercle bacilli, and the removal of diseased tonsils as a preventive to tuberculosis, is probably the most satisfactory thing to do.

The blood supply of the tonsils has been very accurately worked out by numerous investigators. There is no question but that a certain amount of absorption into the system takes place through the blood stream as well as the lymphatics. The blood supply of the tonsils is important on account of the surgery of the tonsil. The time is past when one can ruthlessly remove the tonsil and make no scientific effort to control the blood supply, as in surgical procedure. Severe hemorrhage from the tonsil following a tonsillectomy may be as important a factor in pro ducing shock as the loss of blood from any traumatism or other surgical procedure. Therefore we should aim to secure, either preceding enucleation of the tonsil or immediately following, all bleeding points with the aid of a hemostat. The true tonsilar artery and vein (J. Leslie Davis) pass down behind the tonsil and through the aponeurosis, near the superior and posterior portion of the tonsil, entering the tonsil at its median point.

**Hemorrhage.**

The tonsillar branch of the dorsalis linguae enters near the base of the tonsil about the median line. The point of hemorrhage at this spot as a rule cannot be detected unless the base of the tongue is well depressed. The lingual branch, however, may be found sometimes near the middle of the fossa. Sometimes there is a small bleeding point on the internal wall of the fossa, a branch of the dorsalis inguae,and about the median line and near the attachment of the posterior pillar. These three points are the ones where we anticipate serious hemorrhage.

I want to explain a little later on my method for controlling hemorrhage in tonsillectomy.

**Nomenclature.**

The nomenclature of diseases of the tonsils in their relative frequency are acute and chronic cryptic tonsillitis, acute and chronic interstitial tonsillitis, acute and chronic peritonsillitis, and mebranous tonsilitis. Among the latter are particularly diphtheria and strepococcus infections. Other organisms may produce membranous tonsillitis, but diphtheria and streptococcus infection, more particularly hemolyticus, are the most virulent forms of membranous tonsillitis.

Tuberculosis, syphilis, hyperkeratosis, Vincent-Plaut angina and homerrhagic angina comprise most of the diseases of the tonsils. The hemorrhagic angina not infrequently follows ofter birth, and such cases usually die. Chronic interstitial tonsillitis, and without perceptible local symptoms, is sometimes a factor in producing pain in the ears and pain about the larynx, sometimes radiating to the temples of the diseased side.

**Diagnosis.**

To reiterate the suggestion made, it is very easy to diagnose an acute inflammatory condition involving the tonsils; but a chronic and latent inflammation of the tonsils is very difficult of diagnosis. Sometimes the tonsils are greatly hypertrophied; at other times, so submerged between the pillars as to be totally obscured, and unless by evulsing the tonsil, its absence or presence cannot be detected. A small hypertrophy of the tonsil, except as it prevents good breathing, may not be a source of absorption. A tonsil containing cheesy deposits, constantly or at times, foul and offensive, should be removed. A foul breath is more often from an exposed or hidden crypt than from diseased teeth, or stomach or nasal disorders.

This paper has nothing to do with therapy of tonsillar affections, but has to do briefly with the sequelae of tonsillar diseases and with the surgery of the tonsils.

**Tonsillar Systemic Infections.**

Probably the general practioner is more interestetd in the relationship of tonsils to systemic and focal infections, and no place has there been a closer relationship, as demonstrated by numerous contributions, between acute articular rheumatism and acute tonsillitis. There is a condition that is closely allied to so-called rheumatism, and that is the radiating pains to the neck, arms, fingers and back, which is subject to acute exacerbations. The throat symptoms as a rule precede the articular rheumatism. Endocarditis or pericarditis not infrequently have their origin in the immediate removal of the tonsils, and the disease may be relieved and even cured by the immediate removal of the tonsils. Chorea in young children is sometimes alleviated by removal of the tonsils.

I feel convinced that the tonsils are frequently the avenue of infection in tuberculosis of the lungs; that their removal is not contraindicated in tuberculosis, and that the operation can only be beneficial and not otherwise.

**Hyperthyroidism and the Tonsil.**

There is probably an intimate connection in some cases between diseases of the tonsils and enlargement of the thyroid gland in your children, and diseases of the thyroid in adults, particularly exophthalmic goiter. Dr. B. R. Shurley, Detroit, and others in this country, as well as abroad, have made this point quite clear. Even in advanced cases of exophthalmic goiter I have seen a wonderful alleviation of all the symptoms through the total removal of the diseased tonsils.

In middle-ear disease, either acute serous or catarrhal, in young children, there is usually an amelioration of symptoms following the removal of tonsils and adenoids. Middle-ear deafness is a prevalent condition and in a great majority of cases due to neglect of the nose and throat in infancy. It usually manifests itself in middle life, and is so far developed at that time that treatment is often unsatisfactory.

**Tonsillectomy.**

Danger of operation, where tonsillectomy is indicated, is practically nil; that is, in the hands of one familiar with the technique of the operation. In only one case in many hundreds of operations have we had a death following a tonsillectomy, and this was due to a status lyphaticus, as shown by post-mortem examination. Francis R. Packard, of Philadelphia, and W. Humes Roberts, of Pasadena, California, and many others have reported deaths from status lyphaticus following tonsillectomy under general anesthesia.

We usually have about one severe case of secondary hemorrhage in fifty cases. Secondary hemorrhage may come on in young children as well as adults, and age apparently plays little part in hemorrhage. Paralysis of the palate muscles, with regurgitation of fluids, has been reported by Dunbar Roy, of Atlanta, and others. However, in his case the paralysis passed away after a short time. Not uncommonly have we observed a temporary paralysis of the fauces for a few hours after using a local anesthetic, with regurgitation of fluids into the nose.

### Complications.

Pneumonia is a complication that may follow tonsillectomy, providing the patient is taken out of doors and exposed to dust a few hours after operation, and a patient should remain for at least twenty-four hours in a hospital· The exigencies of the occasion may demand removal in operations done in the physician's office, but after all this is a bad practice.

Tearing of the pillars by an operator, more particularly the posterior pillars, always leads to more or less cicatricial change, producing sometimes a narrowing of the post-nasal space. If narrowing occurs, a horizontal slit should be made through the posterior pillars. If there is a tear in the pillars at the time of operation, the operator should have enough presence of mind to close the perforations. A short time ago a physician in Southern California was defendant in a malpractice suit for fifteen thousand dollars, for accidentally destroying the pillars of the fauces while removing the tonsils. There was was such marked evidence of carlessness or incompetence that the man was compelled to pay a large indemnity, in fact thirty-five hundred dollars.

The voice is seldom materially affected in injury to the pillars and there is little danger of injuring the voice in any case, providing the operation is carefully performed.

### After Treatment.

The after-treatment is the one that seldom receives any attention from the operator. The pain and soreness following a tonsillectomy is usually severe, and in consequence an opiate should be given to relieve pain and distress· Edema of the uvula not infrequently occurs, and this should be relieved by puncturing with a sharp knife. A child or adult should remain in bed for twenty-four hours following an operation. Where a general anesthetic is used and the patient complains of a great deal of thirst, we allow him to take ice water in small quantities. The patient may use a gargle composed of tanic acid and sulphocarbolate of zinc, of each fifteen grains; carbolic acid, five drops; glycerine, two drachms; pepermint water, sufficient quantity to make one ounce.

### The Anesthetic.

For tonsillectomy under general anesthesia we preferably use ether, given by the drop method. Some men order to be given, a half hour before operating, both to children and adults, a small dose of atropin for the prevention of excess of mucus.

For local anesthesia we prefer a one to two per cent. novocaine and adrenalin solution; and in the absence of this, we use one-half of one per cent. cocaine, to which has been added adrenalin ten drops, and two drops of phenol to a dram of one-half per cent. cocaine.

This is first injected beneath the mucous membrane of the anterior pillar, beginning near the base of the tonsil and working up toward the superior fauca, and in the posterior pillar and back of the tonsil. About ten drops of the fluid is also injected deep into the base of the tonsil about the capsule. About thirty to forty drops is all that is necessary to each tonsil.

**Various Operations.**

Methods of operation under local or general anesthesia are many. We have before us a great number of reprints devoted to the tonsil operations · Many of these operations are almost duplicates of each other. Probably there is no operation on the tonsils that has more advocates and notoriety than the Sluder operation. There are only a very men, according to our observation, who are skilled enough to use the Sluder method with satisfaction. The operation which we propose to describe is, in our opinion, very satisfactory.

**A Simplified Operation.**

Under good illumination, if with a general anesthetic, the patient should be in a horizontal plane with the head slightly elevated and mouth opened with a Whitehead gag, preferably with tongue depressor attached. The tonsils should be grasped in the median plane and high up, and the most preferable vulsellum forcep that I know of is Museux'. With the complete or partially submerged tonsil taut, the anterior pillar fits like a glove. With a Seiler's nasal scissors a buttonhole can be made between the tonsil and the anterior pillar, and by gradually pushing the anterior scissors backward and close to the tonsil, the aponeurosis is reached. By separating the scissors a dry dissection of the anterior portion of the tonsil is made. With the tonsil still taut, and with a curved Prince's scissors, if there is any attachment of the posterior pillar superiorly to the tonsil, it may be relieved. With the tonsil still retracted, a long curved hemostat of some make, preferably a Kelly's hemostat, may be gently passed behind the tonsil and between the pillars, and the aponeurosis firmly secured. Within the aponeurosis will be found the true tonsillary artery and vein. After the hemostat has been placed securely in position, and with the Prince's scissors, the aponeurosis that binds, the tonsil superiorly can be cut. The tonsil now hangs pedunculated and a wire snare, preferably a Pierce-Mueller, can be placed about the pedicle. The hemostat still remains in position after the tonsil has been removed. The hemostat in this position, after the tonsil has been removed, acts as a retractor, exposing the fauca to view, and any bleeding points on the pillar or base of the fauca can be easily detected and grasped with an artery forcep. By making gentle pressure with a gauze-tipped sponge holder, bleeding from the capillaries can be easily controlled. Sometimes the artery forceps may be allowed to remain attached to the vessels during the enucleation of the opposite tonsil.

The hemostat should remain attached to any bleeding point three or four minutes. Sometimes even after the removal of the hemostat bleeding may begin, and it is necessary to reapply. By taking these precautions, it is seldom ever necessary to sew the pillars or tampon the fauca.

It is very frequently unnecessary to separate the posterior pillar from the tonsil. The one point to be remembered in a tonsillectomy is that the tonsil itself must be kept perfectly taut during the whole of the enucleation. After enucleation, and all hemorrhage has been absolutely controlled—and this should always be at the operating table—the pillars and fauces should be inspected for possibility of a small amount of tonsillar tissue remaining. The tonsil will not reproduce itself, but sometimes a small amount of lymphoid at the base of the tongue may, by a slow process, work its way upward and between the pillars and resemble true tonsillar tissue, and may even be as great a menace and receptacle for infection as the true tonsillar tissue. —The Medical Council.

---

### THE CONTROL OF SMALLPOX.

Diphtheria, scarlet fever, whooping cough, measles, smallpox, and infantile paralysis are contagions every state desires to control; therefore, quarantines of varying degrees of severity are established by different states.

Quarantine and control as a state proposition is very expensive when state system of control is enforced; even then it frequently fails. Such a proposition can be much easier and effectively handled in cities. Quarantine and control problems and the responsibility is purely local.

In the control of smallpox, laws require notification to the health authorities, exclusion of exposed children from scool, warning placards, vaccination, quarantine and disinfection, with penalties for neglect or refusal to comply with any of the requirements.

To notification there can be no objection; the only objection that can be raised against placarding premises is the injury a sign causes commercial, financial, and social interests, with a possible chance of making a dishonet physician. Since the vital interests of a community are of paramount importance, these objections do not carry weight. The dishonest physician is the only one that would cause anxiety, but they are usually discovered by inspectors or law officers.

The vaccination and the revaccination requirment need no comment, since vaccination has been proved time out of mind to be the surest safe-guard against smallpox. It is not necessary to produce tatistics in the matter; experience will convince where an array of figures will fail. Our personal experience when intimately connected with the management of smallpox, the employment of nurses and others connected with the hospital, shows an absolute immunity against the disease in those who were either vaccinated or recently revaccinated; none others were under employment there.

The violation of the principles of personal liberty, in its relation to vaccination, must not be considered in the control of smallpox in either adult or minor person, for the reason that the advocates of this principle are a menace to a commuinty where they have been exposed as contact or suspect.

The Police and Fire Departments and the Public Schools in the District of Columbia require vaccination or revaccination or evidence of an attack of smallpox, as a prerequisite for appointment or admission. And the cases of this disease among these persons is negligible.

The quarantine requirement is the most important for consideration. It is this that causes more trouble and expense than anything connected with the management of the disease; there is either no intelligent interpretation of the term, or there is an abuse of the power to control, which the law permits.

Quarantine alone does not prevent smallpox after the expiration of the legal period of the quarantine; it only adds to the chances or possibilities of an outbreak of the disease at some future time; it alone is never effective after the appearance of smallpox or where there has been a mild, undiscovered case, as these undiscovered cases, while not ill enough to seek the advice of a physician, have frequented public places, public entertainments, and have been carriers of the infection and contagion.

Quarantine to be of any service must be enforced with vaccination; it is here that quarantine exerts its influence; a quarantine without vaccination will grant a temporary immunity; its effect is local and its only lasting effect is to encourage the unvaccinated to remain unvaccinated; it only makes the offender more strongly impressed in his fallacies. "Effectually quarantined" is an expression too vaguely used; it is a false security, and instead of a city having a protected population, the number of the unvaccinated steadily multiplies; liability to smallpox is retained and increased.

"No health officer or health department that claims that quarantine will stop the occurrence or spread of smallpox has a right to insist upon the people's being vaccinated, for, if quarantine prevents smallpox, it prevents the necessity of vaccination."—(Special Bulletin No. 45, March, 1915, State Board of Health of North Carolina.)—To this should have been added: Nor shall such officer or department be sustained by the state of North Carolina.

Our belief has been and still remains that quarantine is a fallacy unless it is established in connection with vaccination for the unvaccinated. We advocate the vaccination of all who have been exposed to the disease. We advocate and would compel the vaccination and if necessary the revaccination of every unvaccinated contact, with his removal to a municipal quarantine station, and his detention during the legal period of quarantine; revaccinate every previously vaccinated contact and discharge him, but require him to report at stated times for observation. In the event of his refusal to comply with these directions, he, too, would be placed in the quarantine station, but this man would not cause much worry, for did the disease manifest itself, it would soon enough be known. This is not an honor quarantine; it is an intelligent quarantine, for the reason it entails no hardship upon the breadwinner; it reduces the expense of control, and preserves the friendship of the man; he will become a willing and an unpaid assistant to a health department.

He would in the end be able by this method to abolish quarantine against this disease.

Our conclusions are: 1—quarantine is expensive; 2—quarantine without vaccination is a fallacy; 3—quarantine without vaccination begets a feeling of false security; 4—"Effectually quarantined," as employed by the public, is an unworthy expression; 5—vaccination is the only protection against smallpox; 6—abolish quarantine and let the responsibility rest upon those members of the community who want the disease; 7—every city should charge and collect for the maintainance of those sick of the disease, who are, when sick, not residents of the city where they are found; 8—abolish the placard just as soon as disinfection of the premises has been completed; 9—remove evry case of smallpox to a municipal smallpox hospital; 10—levy the cost of home quarantine against the premises; 11—if necessary, refuse supplies at the city's expense.
—The Virginia Medical Semi-Monthly.

